# Supplementary material for: Helix Alignment, Chevrons, and Edge Dislocations in Twist‐Bend Ferroelectric Nematics
Source: Adv Sci (Weinh). 2025 Nov 13:e15752. Online ahead of print. doi: 10.1002/advs.202515752 (PMC13325512; doi:10.1002/advs.202515752)
Supplement: Supplementary file 1 — Supporting Information [file ADVS-9999-e15752-s001.docx]

**Helix alignment, chevrons, and edge dislocations in**

**twist-bend ferroelectric nematics**

Bijaya Basnet^1,2^, Priyanka Kumari^1,2^, Sathyanarayana Paladugu^1^, Damian Pociecha^3^, Jakub Karcz^4^, Przemysław Kula^4^, Nataša Vaupotič^5,6^, Ewa Górecka^3^, and Oleg D Lavrentovich ^1,2,3,7*^

*^1^Advanced Materials and Liquid Crystal Institute, Kent State University, Kent, OH 44242, USA*

*^2^Materials Science Graduate Program, Kent State University, Kent, OH 44242, USA*

*^3^Faculty of Chemistry, University of Warsaw, Zwirki i Wigury 101, Warsaw 02-089, Poland*

*^4^Faculty of Advanced Technology and Chemistry, Military University of Technology, Warsaw, Poland*

*^5^Jozef Stefan Institute, Jamova 39, 1000 Ljubljana, Slovenia*

*^6^Department of Physics, Faculty of Natural Sciences and Mathematics, University of Maribor, Koroška 160, 2000 Maribor, Slovenia*

*^7^ Department of Physics, Kent State University, Kent, OH 44242, USA*

*Authors for correspondence: e-mails: [olavrent@kent.edu](mailto:olavrent@kent.edu)


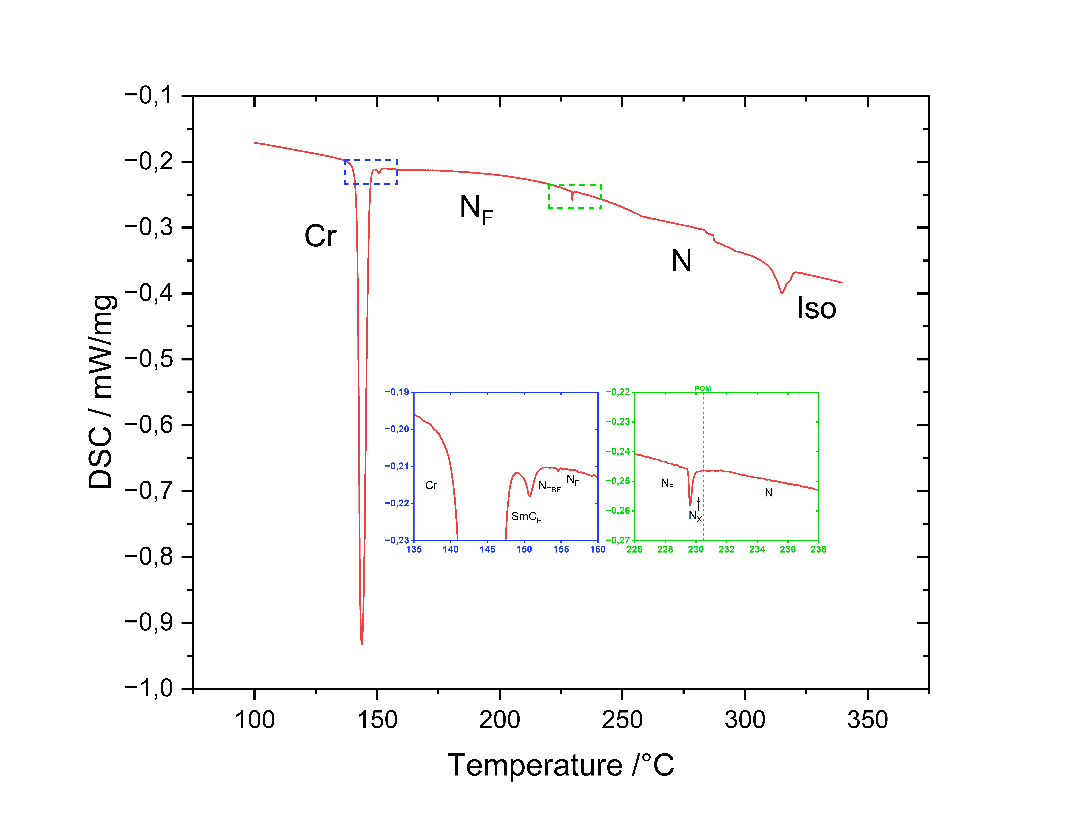


Figure S1: Differential Scanning Calorimetry (DSC) for JK203. DSC thermogram recorded on heating (10 K/min).

**
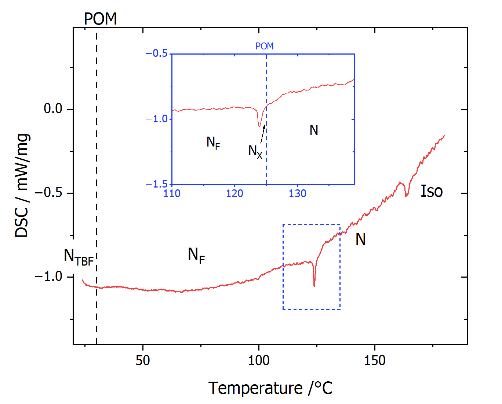
**

Figure S2: DSC for NTBF005. DSC thermogram recorded on heating (10 K/min). Dashed lines show N_TBF_-N_F_ and N_X_-N transition temperatures determined from optical studies.


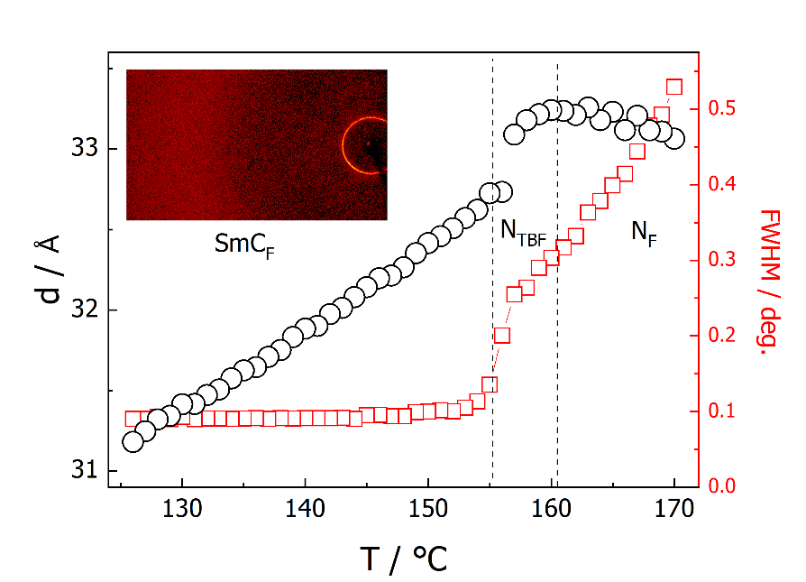


Figure S3: X-ray diffraction (XRD) for JK203. Layer spacing in the smectic phase and local periodicity detected in nematic phases of JK203 by small angle X-ray diffraction (open circles); full width at half maximum (FWHM) of the diffraction signal (red squares) showing gradual development of positional order in nematic phases, which in smectic phase becomes truly long-range (the width of the XRD signal related to density periodicity is machine resolution limited). The inset shows a 2D XRD pattern recorded in wide angle range in the SmC_F_ phase.


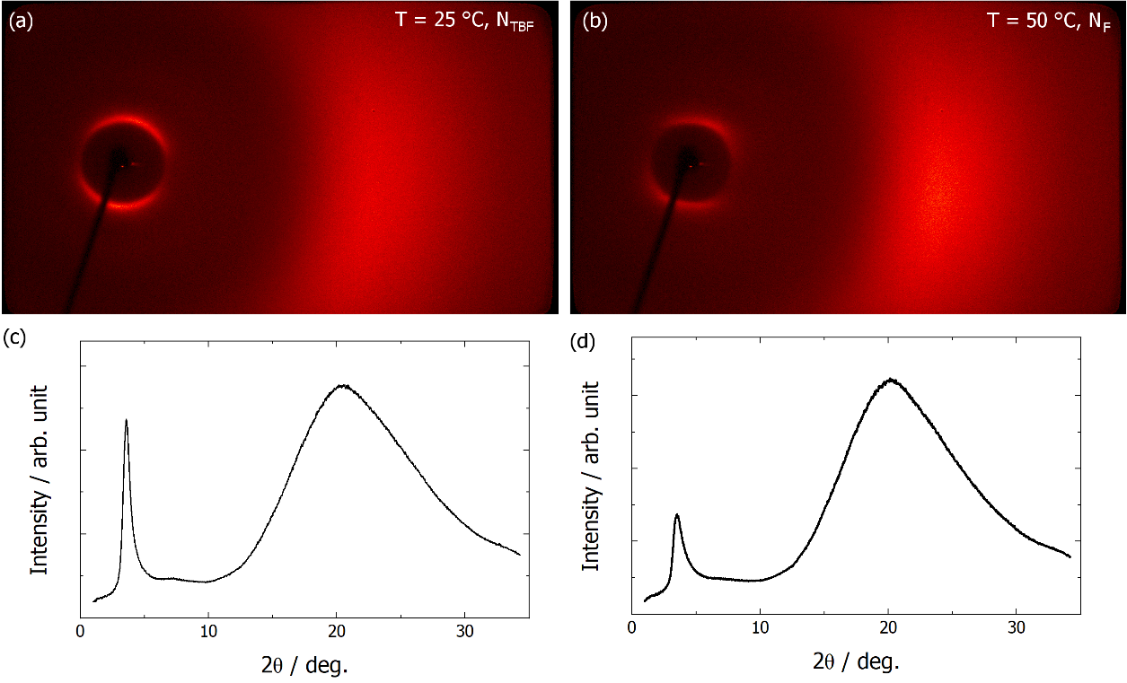


Figure S4: XRD for NTBF005. 2D XRD patterns recorded in wide angle range in (a) N_TBF_ and (b) N_F_ phases. (c) and (d) Diffracted intensity vs. diffraction angle obtained by integration over the azimuthal angle of the patterns presented in (a) and (b), respectively. In both phases all diffraction signals are broadened with respect to instrumental resolution, reflecting lack of long-range positional order of molecules.

Figure S5: Electric circuit to measure the polarization density from the polarization reversal produced by an in-plane electric field. The ITO-glass substrates of 100 Ω/$◻$ resistance were patterned lithographically to produce 2 ITO electrode stripes (2 mm × 14 mm) on the 11 mm × 16 mm glass area. The electrodes are separated by a 1 mm gap. The ITO-glass plates are covered with a polyimide layer which is rubbed to set the surface alignment collinear with the in-plane electric field. An in-plane ac electric field of a triangular waveform, frequency 50 Hz, and peak-to-peak voltage of 240 V was applied using a Siglent SDG1032X waveform generator and an amplifier (Krohn-Hite corporation). Cells of a gap thickness $d=$ (20.0 $\pm$ 0.1) µm are used. The electric current through a 20 k Ω resistor is measured with an oscilloscope Tektronix TDS 2014 (sampling rate 1GSa/s). When the polarity of the triangular wave is reversed, the net polarization charge produced by the polarization reversal is $Q=2PA$, where $A$ = 20 μm × 1.4 cm is the cross-sectional area of the liquid crystal. The polarization density is calculated as $P=\frac{\int I\left( t \right) dt}{2A}$ where $\int I\left( t \right) dt$ is the area under the polarization current curve. A similar set-up is used to verify the polarization alignment with respect to the rubbing direction; in this case, thin cells, $d=$ (2.3 $\pm$ 0.1) µm, are used to avoid twist deformations.

Figure S6: Polarization density measurements for a) JK103, b) JK203, and c) NTBF005. The time dependence of applied voltage (triangular waveform, $f=$ 50 Hz and peak-to-peak voltage 240 V) and electric current. Thicker black solid line represents the applied voltage whereas thinner solid lines represent the electric currents at different temperatures. $d=$ (20.0 $\pm$0.1) µm; $A$ = 20 μm × 1.4 cm.

Figure S7: Polarization density of DIO as a function of temperature: a) time dependence of applied voltage (triangular waveform, $f=$ 50 Hz and peak-to-peak voltage 240 V) and electric current. Thicker black solid line represents the applied voltage whereas thinner solid lines represent the electric currents at different temperatures; b) The polarization density as a function of temperature in the $N_{F}$ phase. $d=$ (20.0 $\pm$0.1) µm; $A$ = 20 μm × 1.4 cm.

Figure S8: Polarizing microscope textures of a uniform polarization alignment in thin planar N_F_ cell of JK103. a-c) A direct current (dc) electric field antiparallel to the rubbing direction **R** (**E**$\uparrow$**R**$\downarrow$) does not change the interference color while application of the field along **R** (**E**$\downarrow$**R**$\downarrow$) rotates $\boldsymbol{P}$ counterclockwise and clockwise, producing domains 1 and 2, respectively; d-f) the same, observations with a full-wavelength 550 nm optical compensator. $d=$ (2.3 $\pm$ 0.1) µm; 120 $℃$. The electric field response illustrates that **P** is antiparallel to the buffing direction **R**.
